# The fungal pathogen *Rhizoctonia solani* AG-8 has 2 nuclear haplotypes that differ in abundance

**DOI:** 10.1093/g3journal/jkaf252

**Published:** 2025-10-23

**Authors:** Jana Sperschneider, Kathleen DeBoer, Karam B Singh, Gupta V S R Vadakattu, Jonathan Anderson

**Affiliations:** CSIRO Agriculture and Food, Canberra, ACT 2601, Australia; CSIRO Agriculture and Food, Floreat, WA 6010, Australia; CSIRO Agriculture and Food, Floreat, WA 6010, Australia; The UWA Institute of Agriculture, The University of Western Australia, Crawley, WA 6009, Australia; CSIRO Agriculture and Food, Waite, SA 5064, Australia; CSIRO Agriculture and Food, Floreat, WA 6010, Australia; The UWA Institute of Agriculture, The University of Western Australia, Crawley, WA 6009, Australia

**Keywords:** *Rhizoctonia solani AG8*, genome assembly, heterokaryotic fungus, haplotype phasing, plant-pathogen interactions

## Abstract

The fungal pathogen *Rhizoctonia solani* infects a diverse range of host plants and remains an intractable and economically significant disease for many crops. *R. solani* is classified into reproductively incompatible anastomosis groups (AGs). In the vegetative stage, most plant-pathogenic *R. solani* isolates are multinuclear and heterokaryotic, but little was previously known about the diversity between haplotypes due to highly fragmented, collapsed short-read assemblies. We present fully-phased, chromosome-scale genome assemblies of the broad host-range *R. solani* isolates AG8-1 and AG8-3. We demonstrate that both AG8 isolates have 2 distinct haplotypes, each of which is ∼50 Mbp spread across 16 chromosomes and use PacBio Iso-Seq data to achieve a high-quality gene annotation. We show that the 2 nuclear haplotypes display high heterozygosity and differences in haplotype abundance in vegetative cultures. Using transcriptome sequencing during infection of different host plants for AG8-1 and wheat for AG8-3, we show that the less abundant haplotype in both AG8-1 and AG8-3 might harbor more genes upregulated during infection. Taken together, these findings address some of the observed phylogenetic heterogeneity of AG-8 isolates and provide a platform to further dissect the mechanisms enabling this globally significant agricultural pathogen to inflict losses to a range of crop hosts.

## Introduction

*Rhizoctonia solani*, a fungal species complex encompassing both pathogenic and nonpathogenic forms, is a significant threat to forestry and agriculture worldwide (Nagaraj et al. 2017). *R. solani* is responsible for a range of diseases, including root, crown, and stem rot, as well as damping-off and wilting, affecting a wide variety of economically important crops (Anderson 1982; Nizamani et al. 2025). *R. solani* is a fungal species complex comprising multiple genetic groups known as anastomosis groups (AGs), which are classified based on their ability to anastomose, or fuse, with hyphae of isolates belonging to the same group. Currently, 13 distinct AGs have been identified, along with a group of bridging isolates, AG-BI (Carling et al. 2002). These AGs differ in various aspects, including morphological traits and host range and can be distinguished at the genetic level, further supporting their classification as separate subspecies (Sneh et al. 1996). *R. solani* AG-8 is the causal agent of Rhizoctonia root rot and bare patch of wheat and barley. AG-8 isolates typically possess a broad host range, attacking both cereals and dicot crops including those commonly rotated with wheat or barley such as chickpea, lentil, lupin, and canola (Sweetingham et al. 1986; Khangura et al. 1999). Isolates of the AG-8 group result in losses of approximately $137 million annually in Australian cereal production (Murray and Brennan 2010; Huberli et al. 2025), over $100 million in Washington state, and billions of dollars globally (Okubara et al. 2014). While chemical control options exist in some crops, the availability of effective and reliable agrochemicals is limited, thereby necessitating the adoption of alternative management strategies. Currently, crop protection largely relies on practices such as crop rotation, tillage, and, where possible, the use of resistant crop varieties (Okubara et al. 2014). These approaches are vital in mitigating the impact of *R. solani* and improving crop resilience and production against this widespread pathogen.

In natural environments, *R. solani* primarily reproduces asexually, existing mainly as vegetative mycelium or sclerotia, which serve as resting structures that enhance its survival and persistence in soil and plant debris (Anderson 1982). Isolates within the same AG may be able to form heterokaryons resulting from the exchange of genetic material during anastomosis (Bolkan 1974). As a result, the AGs of *R. solani* can largely be considered reproductively isolated, each functioning as a distinct subspecies with specific ecological niches and pathogenic capabilities. Many of the highly pathogenic isolates of *R. solani* are multinuclear heterokaryotes, maintaining separate nuclei containing substantial diversity between the haplotypes. The heterokaryotic state creates difficulties in assembling genomes and assigning reads, particularly short reads, to any one individual haplotype. Thus, genomes assemblies from multinucleate isolates to date have mostly assembled haploid representations of the heterokaryotic state.

Currently, 24 genome assemblies exist for *R. solani* with isolates originating from AG1-1, AG2-2, AG-3, AG-4, AG-6, and AG-8 (Cubeta et al. 2014; Hane et al. 2014; Wibberg et al. 2015, 2016, 2017; Nadarajah et al. 2017; Ghosh et al. 2019; Li et al. 2021; Zhang et al. 2021; Kaushik et al. 2022; Lu et al. 2023; Liu et al. 2024; Xu et al. 2024). These draft genome assemblies exhibit a remarkable size range, with estimates from 33 Mbp for an AG1-IC isolate to approximately 70 Mbp for the AG3-1A1 and AG2-2IIIB consensus haploid assemblies (Wibberg et al. 2016; Kaushik et al. 2022). The diploid assembly of the uninucleate AG1-IA JN isolate was the largest to date at 97 Mbp (Li et al. 2021). This genomic variation underscores the complexity of *R. solani* at the molecular level and highlights the challenges inherent in studying its diverse genomic architecture. The genome data reported herein is the first chromosome-level assembly of haplotype genomes from multinuclear heterokaryotic *R. solani* isolates, which enables accurate analysis of intra-isolate diversity and the involvement of the homoeologous genes in the infection process across the broad range of hosts infected by AG-8.

## Methods and materials

### Growth of fungal isolates and inoculation of plant hosts

The *R. solani* isolate AG8-1 (WAC10335) was described previously (Hane et al. 2014). The AG8-3 isolate (WAC9760) was originally isolated from “bare patch” affected wheat in Esperance Downs, Western Australia (MacNish and Sweetingham 1993) and was supplied by The Department of Primary Industries and Regional Development, Western Australia. Initial cultures were grown on potato dextrose agar (PDA) at 25 °C for 5 days. For inoculation of plant hosts, a plug of agar from PDA cultures of AG8-1 and AG8-3 was used to inoculate sterile millet seed and incubated at 24 °C for 14 d. Millet cultures were dried in the laminar flow for 24 h prior to freezing at −80 °C until use. Five millet seeds were used to inoculate moist soil in 0.8 L pots, and the pots were incubated at 24 °C for 7 d. Noninoculated control pots were treated similarly without the addition of infected millet seeds. Four wheat (cv. Wyalkatchem), narrow leaf lupin (c.v. Coyote), or canola (cv. Zircon) seeds were planted into each pot to a depth of 25 mm and incubated at 16 °C with 12 h of light per day at 350 lum. Plants were scored and photographed at 35 d after inoculation. For RNA-seq analysis of AG8-1 and AG8-3 infections of wheat, *Medicago truncatula*, *Brassica napus*, and *Arabidopsis thaliana* vermiculite was preinfected with 4 Rhizoctonia-infected millet seed per pot for 1 wk at 24 °C prior to planting. Wheat (cv Chinese Spring), *M. truncatula* (A17), and *Brassica napus* (cv Westar) seeds were surface sterilized with 70% ethanol, rinsed in sterile water, and germinated on moist filter paper at 4 °C for 4 d prior to planting into *R. solani* preinfected or noninfected (control) pots. *Arabidopsis thaliana* (Col-0) seeds were grown in vermiculite at 21 °C for 10 d prior to transplanting to *R. solani* preinfected or noninfected (control) pots. All pots were then incubated at 16 °C for 7 d prior to the collection and washing of root tissue. Tissue was immediately frozen in liquid nitrogen and stored at −80 °C until RNA extraction.

### Isolation of genomic DNA, RNA, and sequencing

A portion of hyphae from the growing tip of the colony was transferred to a liquid defined minimal medium (Solomon et al. 2004) with gentle shaking at 75 rpm at 22 °C for 10 d to enable growth of vegetative fungal hyphae in vitro. Hyphal material was recovered from the culture by filtering through sterile cheesecloth and rinsing with sterile water prior to drying with sterile blotting paper. The hyphal material was frozen in liquid nitrogen and stored at −80 °C until DNA extraction. High molecular weight DNA was purified according to a protocol including the precipitation of small fragments using polyethylene glycol (PEG) (Debler et al. 2020). The purified DNA was subjected to PacBio HiFi sequencing by AGRF (Brisbane, Australia). For Hi-C sequencing, mycelium from liquid defined minimal medium cultures was cut into small fragments and cross-linked with 1% formaldehyde in PBS at room temperature for 20 min. Glycine was added to 125 mM final concentration and incubated for 15 min prior to precipitation by centrifugation, washing in PBS and grinding in liquid nitrogen. Samples were sequenced by Phase Genomics (Seattle, USA). PacBio Iso-seq long read RNA sequencing data were obtained from vegetative mycelial cultures grown in potato dextrose broth (PDB) at 23 °C for 7 d with shaking at 75 rpm. RNA was extracted according to Anderson et al. (2017) and sequenced by AGRF (Brisbane, Australia). Stranded RNA-seq of vegetative mycelium and infected plant tissue was extracted (Anderson et al. 2017) and sequenced by Novogene (Hong Kong).

### Genome assembly and haplotype comparisons

The HiFi reads were downsampled with seqkit sample (−proportion 0.5) (Shen et al. 2016) and then assembled using hifiasm 0.19.6 in Hi-C integration mode and with default parameters (Cheng et al. 2021). Contaminants were identified using sequence similarity searches (BLAST 2.11.0 -db nt -evalue 1e-5 -perc_identity 75) (Altschul et al. 1990). HiFi reads were aligned to the assembly with minimap2 v2.22 (-ax map-hifi –secondary = no) (Li 2018), and contig coverage was called using bbmap's pileup.sh tool on the minimap2 alignment file (http://sourceforge.net/projects/bbmap/). All contaminant contigs, contigs with less than 15× coverage, and the mitochondrial contigs were removed from the assembly. Chromosomes were curated using visual inspection of Hi-C contact maps produced using Hi-C-Pro 3.1.0 (MAPQ 10) (Servant et al. 2015) and Hicexplorer 3.7.2 (Ramírez et al. 2018). The 2 haplotypes were compared to each other with mummer 4.0.0rc1, using nucmer and dnadiff (Marçais et al. 2018). Haplotype abundance was estimated by mapping the PacBio HiFi reads to the assemblies with minimap2 (-ax asm20 –secondary=no) and calculating coverage with bbmap's pileup.sh (http://sourceforge.net/projects/bbmap/).

### Annotation and RNA-seq analysis

De novo repeats were predicted with RepeatModeler 2.0.2a and the option -LTRStruct (Flynn et al. 2020), and RepeatMasker 4.1.2p1 (-s -engine ncbi) (http://www.repeatmasker.org) was run with the RepeatModeler library to obtain statistics about repetitive element content. For gene annotation, RNA-seq reads were cleaned with fastp and default settings (Chen et al. 2018) and aligned with STAR 2.7.9a in 2-pass mode (-alignIntronMin 5 –alignIntronMax 3000 –alignMatesGapMax 3000 –outFilterMultimapNmax 100), which removes junctions supported by ≤ 2 reads (Dobin et al. 2013). Transcripts were assembled from the alignment with Stringtie 2.2.1 (-s 1 -m 200) with the appropriate strand setting (Pertea et al. 2015). Iso-Seq reads were cleaned with isoseq refine (https://github.com/PacificBiosciences/pbbioconda) and fastp (–trim_poly_x –adapter_fasta) (Chen et al. 2018). Clean Iso-Seq reads were aligned with minimap2 2.25 (-ax splice:hq -uf -G 3000) and transcripts were assembled with Stringtie (-L -m 200). The resulting sets of RNA-seq and Iso-Seq transcripts were merged into a consensus set with Stringtie (–merge). CodingQuarry 2.0 was run in pathogen mode with these transcripts (Testa et al. 2015). Funannotate 1.8.13 (Palmer and Stajich 2020) was run in training mode with the RNA-seq reads and Iso-Seq transcripts as input. We then ran funannotate predict (–ploidy 2 –optimize_augustus –busco_seed_species ustilago) and supplied the CodingQuarry predictions with the option -other_gff and set the weight to 1. Funannotate iprscan (Jones et al. 2014) and funannotate annotate were used for functional annotation of genes. We predicted secreted proteins as those that have a signal peptide (SignalP 4.1 -u 0.34 -U 0.34) and no transmembrane domains outside the N-terminal signal peptide region (TMHMM 2.0) (Krogh et al. 2001; Petersen et al. 2011). Effectors were predicted from the secreted proteins with EffectorP 3.0 (Sperschneider and Dodds 2022). BUSCO completeness was assessed on the annotated proteins using version 5.4.7 (Simão et al. 2015). For gene expression analysis, we used Salmon 1.10.1 in genome decoy mode (Patro et al. 2017). We used Tximport (type = “salmon”) and DESeq2 to assess gene differential expression following default settings (*P*_adj_ < 0.1) (Love et al. 2014). Orthofinder 2.5.4 was used to extract 1-1 orthologs. We used DGenies and KaryoploteR for visualization of the chromosomes (Gel and Serra 2017; Cabanettes and Klopp 2018)

### Phylogenetic tree

We downloaded all publicly available *Rhizoctonia solani* genome assemblies from NCBI and used PHAME 1.0.5 (Shakya et al. 2020) to generate a maximum likelihood phylogeny which was visualized in iTOL (Letunic and Bork 2007).

## Results and discussion

### Chromosome-scale, nuclear-phased assemblies for *Rhizoctonia solani* AG8-1 and AG8-3

**Fig. 1.**
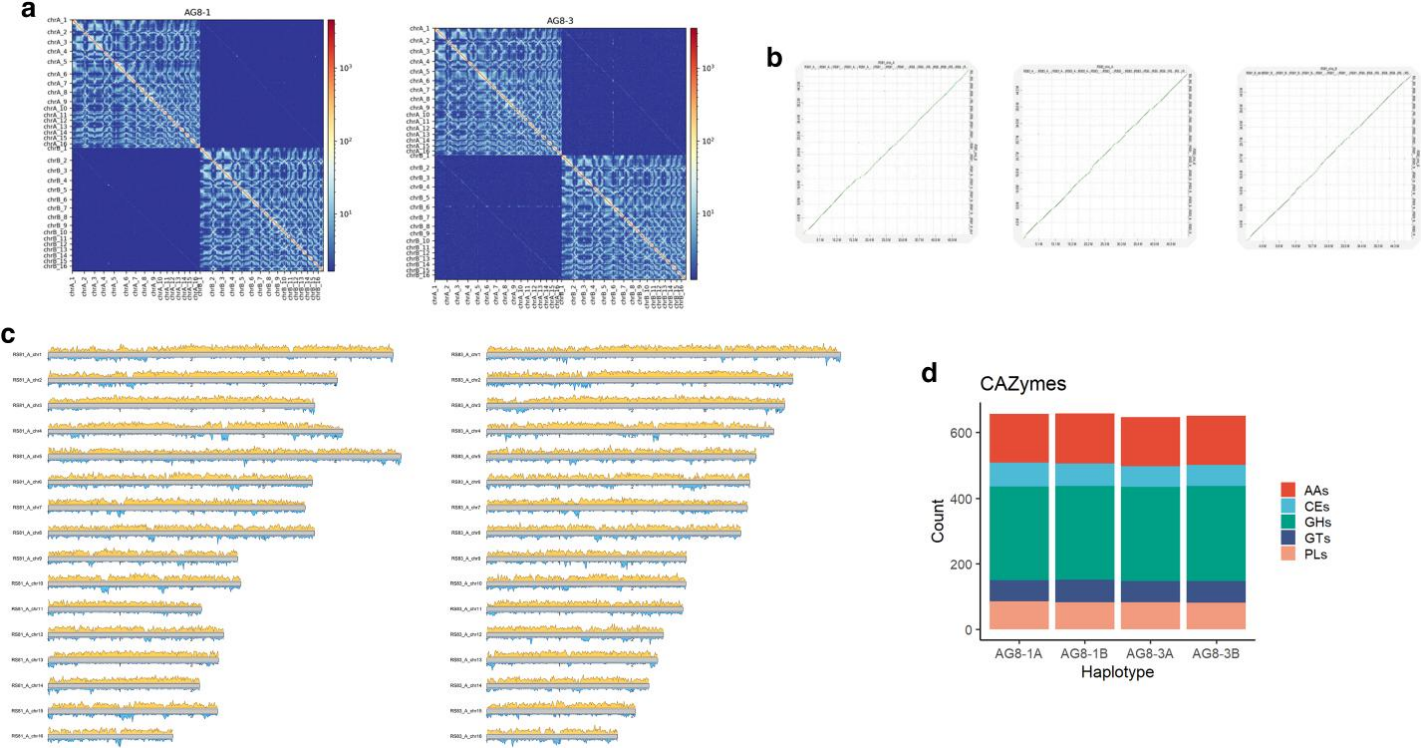
Hi-C contact maps and synteny of the 8-1 and 8-3 chromosomes. a) Hi-C contact maps of isolates 8-1 and 8-3 show 2 distinct nuclear compartments. b) Dot plots show that the chromosomes of the isolates are highly syntenic. c) Gene density and repeat densities (10Kb bins) are shown in yellow and blue, respectively, for one haplotype of AG8-1 and AG8-3. d) Annotated CAZyme content for the 2 isolates and their haplotypes shows uniformity in enzymatic gene content for the 2 haplotypes (AAs: auxiliary activities; CEs: carbohydrate esterases; GHs: glycoside hydrolases; GTs: glycosyl transferases; PLs: polysaccharide lyases).

*R. solani* AG8 has been reported to carry multiple nuclei per cell with thus far unknown ploidy. To resolve its haplotype genomes, we sequenced the broad host-range *R. solani* isolate AG8-1 (WAC10335) and another isolate, AG8-3. In agriculture, both AG8-1 and AG8-3 cause bare patch and root-rot diseases on cereals and legumes with AG8-1 also impacting the survival of canola seedlings (Sweetingham and MacNish 1991; Anderson et al. 2013; Foley et al. 2016) (Supplementary Fig. 1). AG8-1 (formerly known as ZG1-1) also causes root and hypocotyl rot on the model legume *Medicago truncatula* while the model brassica, *Arabidopsis thaliana*, displays root rot symptoms to AG8-1 infection (Perl-Treves et al. 2004; Anderson et al. 2013; Kidd et al. 2021). For both isolates, we used PacBio HiFi and Hi-C data with hifiasm (Cheng et al. 2021) to assemble the genomes (AG8-1: 13.1 Gb HiFi reads and 16.5 Gb Hi-C reads; AG8-3: 12.2 Gb HiFi reads and 12.3 Gb Hi-C reads). This resulted in 2 haplotype assemblies of ∼50 Mbp each (Supplementary Table 1), which we scaffolded into 16 chromosomes per haplotype (Table 1). For AG8-3, 2 contigs displayed phase switches, which we corrected prior to scaffolding. In the AG8-1 assembly, haplotype A contains only a single gap, whereas haplotype B was assembled completely from telomere to telomere (Table 1). In contrast, the AG8-3 assembly has 5 gaps in haplotype A and 2 in haplotype B. The unplaced contigs comprise only ∼1.7 Mbp per isolate and are lower in GC content. Hi-C contact maps of the curated chromosomes display a clear nuclear phasing signal (Fig. 1a and b). The 16 chromosomes are highly collinear, both within each isolate and between the 2 isolates (Fig. 1c, d, and e). These assemblies are a vast improvement compared to the short-read assembly of isolate AG8-1, which was a highly fragmented, haploid representation of the 2 haplotypes (39.8 Mbp, 857 scaffolds with 13.1% gaps, L50:160.5 Kbp) (Hane et al. 2014).

**Table 1.** Genome assembly statistics for *R. solani* AG8-1 and AG8-3.

	AG8-1			AG8-3		
	Haplotype A	Haplotype B	Unplaced contigs	Haplotype A	Haplotype B	Unplaced contigs
Assembly size	51.025 Mbp	49.182 Mbp	1.688 Mbp	50.580 Mbp	49.486 Mbp	1.653 Mbp
No. of scaffolds	16	16	17	16	16	24
Number of gaps	1	0	0	5	2	0
GC content	48.45%	48.47%	43.83%	48.43%	48.44%	41.78%
No. of telomeres (forward/reverse)	12/14	15/14	2/0	13/13	15/14	1/2
Annotated genes	14,941	14,594	217	15,260	15,159	171
Complete BUSCOs	98.8%	99.3%	0.1%	97.7%	97.5%	0.3%
Duplicated BUSCOs	0.9%	0.5%	0.1%	0.7%	0.5%	0%
Genes encoding secreted proteins	1,622 (10.9%)	1,628 (11.2%)	5 (2.3%)	1,647 (10.8%)	1,636 (10.8%)	10 (5.9%)
Predicted secreted effector proteins	516 (31.8%)	517 (31.8%)	1 (20%)	497 (30.2%)	510 (31.2%)	4 (40%)
Repeat content	12.81%	13.24%	19.09%	13.96%	13.21%	24.75%
Retroelements	10.44%	11.03%	11.81%	11.89%	11.34%	14.21%
DNA transposons	1.80%	1.63%	4.06%	1.42%	1.32%	2.04%

Next, we annotated repetitive elements for each genome. All 4 haplotypes had ∼13% repetitive bases, with retroelements the dominant class of transposable elements (Table 1). We observed highly repetitive regions on each chromosome, some of which might correspond to centromeric regions (Fig. 1c). To enable a highly accurate gene annotation, we generated PacBio Iso-seq data for both isolates from vegetative cultures and supplemented this with RNA-seq data from infection (Supplementary Table 2). This resulted in ∼15k genes per haplotype with over 98% BUSCO completeness (Table 1, Supplementary Data 1). We added functional annotations to the gene sets as well as secretome and effector predictions. For AG8-1 and AG8-3, ∼66% of annotated genes have a predicted domain annotation or functional annotation, suggesting a high-quality gene annotation (Supplementary Data 1), and ∼11% of the annotated genes encode predicted secreted proteins (Table 1). Of the secreted proteins, ∼31% are predicted effectors (Table 1), and in line with other necrotrophic pathogens, EffectorP 3.0 predicts 64.2% of these as apoplastic effectors (Sperschneider and Dodds 2022). The A and B haplotypes of AG8-1 have 671 and 670 annotated CAZyme (carbohydrate-active enzyme) genes, respectively, and the A and B haplotypes of AG8-3 have 658 and 664 annotated CAZyme genes, respectively, with equal proportions of CAZyme classifications on the haplotypes (Supplementary Data 1, Fig. 1d). Lastly, the STE3 and HD domain genes that are suggested to be involved in mating are correctly annotated on chromosome 16 and chromosome 11, respectively (Supplementary Data 1) (Li et al. 2021).

### Both *Rhizoctonia solani* AG8-1 and AG8-3 have 2 nuclear haplotypes that are highly heterozygous

**Fig. 2.**
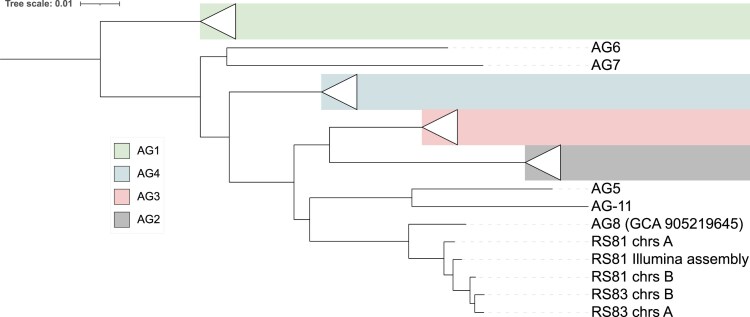
A phylogenetic tree of Rhizoctonia isolate genomes. Rhizoctonia isolates group according to their AG groups (RS81 chrs A = AG8-1 haplotype A; RS81 chrs B = AG8-1 haplotype B; RS83 chrs A = AG8-3 haplotype A; RS83 chrs A = AG8-3 haplotype B; chrs = chromosomes).

Next, we investigated the differences between the 2 nuclear haplotypes for each isolate (Table 2). The haplotypes of isolate AG8-1 are highly heterozygous with ∼9% unaligned bases, 96.8% average identity of alignments, and 880 K SNPs. The haplotypes of isolate AG8-3 are less heterozygous but still substantially different, with ∼7% unaligned bases, 98% average identity of alignments and 551 K SNPs. Interestingly, the AG8-1 haplotype A is substantially different to all other 3 haplotypes, whereas the AG8-1 haplotype B is more similar to the AG8-3 haplotypes than to the AG8-1 haplotype A (Table 2). A phylogenetic tree of publicly available *Rhizoctonia* genome assemblies confirms that the AG8-1 haplotype B is more closely related to the AG8-3 haplotypes than to the AG8-1 haplotype B (Fig. 2). Furthermore, the placement of the short-read assembly of AG8-1 in the phylogenetic tree confirms that it is a collapsed representation of the 2 haplotypes (Hane et al. 2014). We further investigated the SNP content between the 2 haplotypes to assess if repeat-induced point (RIP) mutations are present, as indicated previously (Hane et al. 2014). Using the RIPper software (van Wyk et al. 2019), only 0.07 and 0.09% of the AG8-1 and AG8-3 genomes were estimated to be affected by RIP, respectively. However, it may be possible that a RIP-like process might operate in *Rhizoctonia* that is different from the one in ascomycetes.

**Table 2.** Haplotype alignment and SNP statistics.

SNPs|alignment identity	AG8-1A	AG8-1B	AG8-3A	AG8-3B
AG8-1A	-	880,333 | 96.82%	890,832 | 96.77%	902,196 | 96.74%
AG8-1B		-	551,518 | 98.11%	557,047 | 98.09%
AG8-3A			-	551,138 | 98.11%
AG8-3B				-

### Haplotype abundance and transcriptome contribution of coexisting genomes during plant infection

**Fig. 3.**
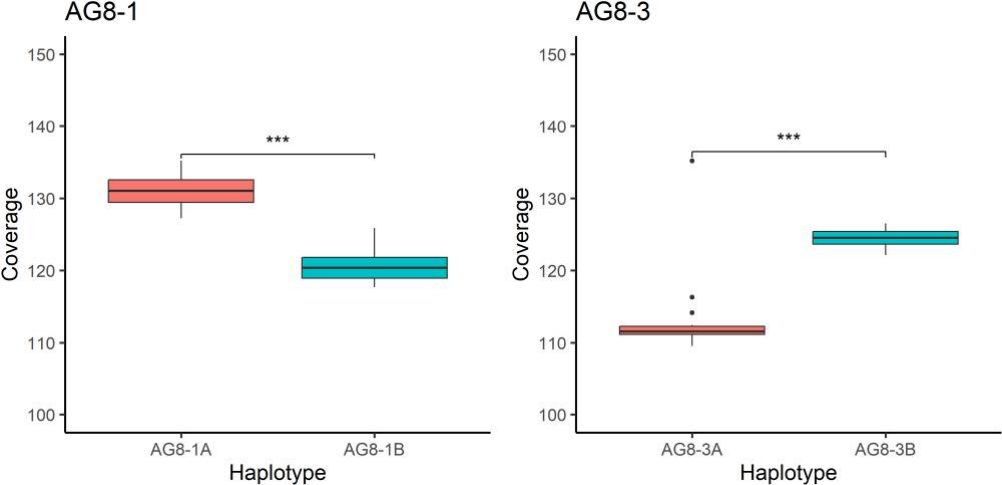
Haplotype abundance indicated by chromosome read coverage for the 2 isolates.

Mapping of the PacBio HiFi reads back to the assemblies indicated that the 2 haplotypes occur at significantly different abundances in vegetative cultures (Fig. 3). For AG8-1, the A haplotype is more abundant than the B haplotype (8.5% higher). In contrast, for AG8-3, the B haplotype is more abundant than the A haplotype (9.9% higher). To compare gene expression between the 2 haplotypes, we conducted a protein orthology analysis. We selected single-copy orthologs that differentiated by at least 1 SNP in their coding sequences to assess gene expression on the 2 haplotypes (AG8-1: *n =* 10,514; AG8-3: *n =* 9,873). We used RNA-seq data from infection of the natural host *Triticum aestivum* with AG8-1 or AG8-3 as well as infection of the natural host *Brassica napus* and genetically tractible model plant species *Arabidopsis thaliana* (a member of the brassica family) and *Medicago truncatula* (a member of the legume family) with AG8-1 (Supplementary Table 2, Supplementary Data 1). Principal component analysis (PCA) of RNA-seq data shows that all samples cluster according to biological conditions (Supplementary Fig. 2).

**Fig. 4.**
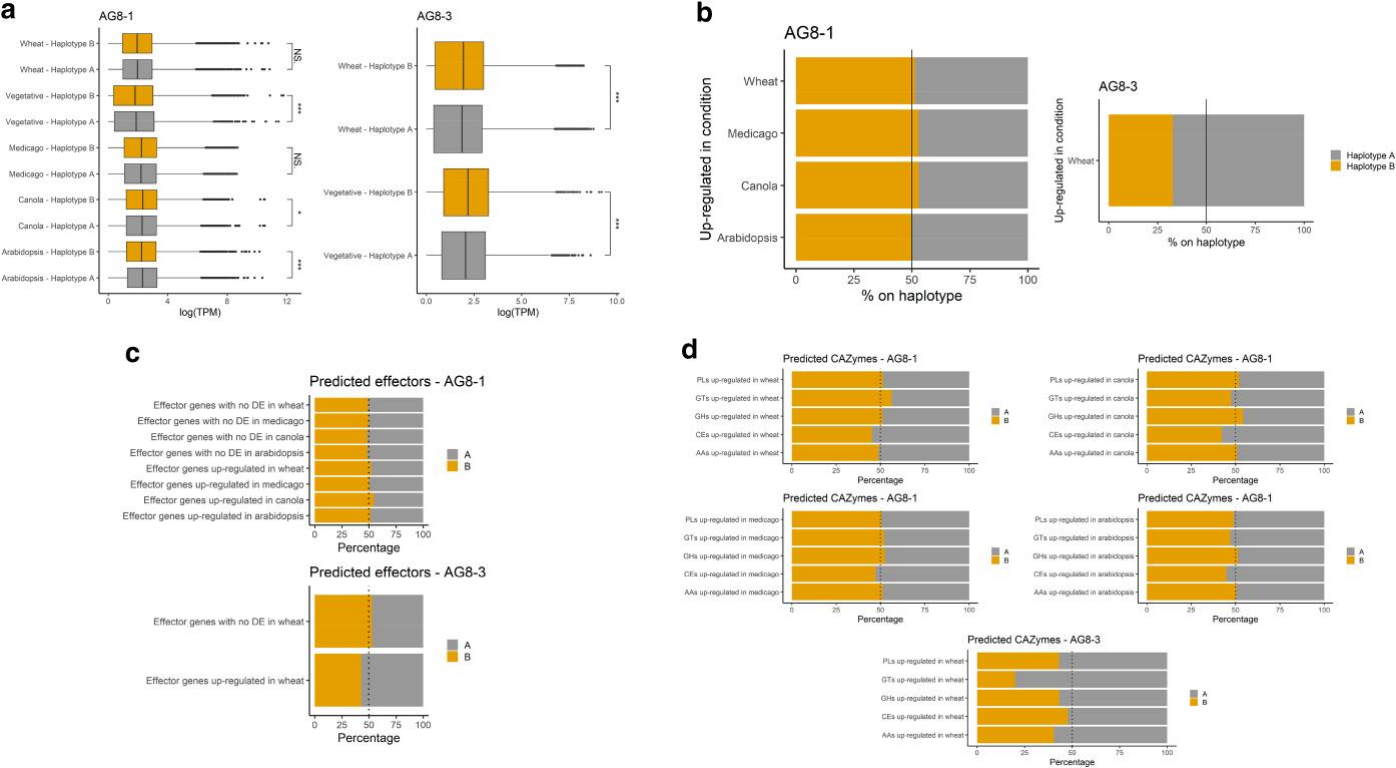
RNA-seq expression analysis during vegetative stages and host infection. a) Significantly higher TPM values are recorded for the more abundant haplotype for AG8-1 (haplotype A) and AG8-3 (haplotype B) in most conditions. b) In contrast, a higher proportion of genes upregulated during plant infection reside on the A haplotype with lower abundance in AG8-3. The AG8-1 isolate showed a slight over-representation of genes upregulated during plant infection from the less abundant B haplotype during infection of wheat, Medicago, and canola. c) The less abundant haplotypes for AG8-1 (haplotype B) and AG8-3 (haplotype A) carry more upregulated genes encoding predicted effectors during infection of wheat (AG8-1 and AG8-3), canola (AG8-1), and *Arabidopsis* (AG8-1). DE stands for differentially expressed. d) The proportions of CAZymes that are upregulated during infection are shown for the 2 haplotypes (AAs: auxiliary activities; CEs: carbohydrate esterases; GHs: glycoside hydrolases; GTs: glycosyl transferases; PLs: polysaccharide lyases).

When comparing transcription between haplotypes, for AG8-1, the more abundant A haplotype has significantly higher transcription than the B haplotype in the vegetative state and during *Arabidopsis thaliana* infection but not during wheat, canola (*Brassica napus*), and barrel medic/medicago (*Medicago truncatula*) infection (Fig. 4). Similarly, the more abundant B haplotype has higher transcription than the A haplotype for AG8-3 (Fig. 4). However, gene regulation does not follow the same trend of haplotype abundance. In AG8-3, 67.1% of genes upregulated during infection of wheat are located on the less abundant A haplotype (Fig. 4), suggesting a potential for greater involvement of the genes contained within this haplotype to be important for infection of wheat. The AG8-1 isolate showed a lower degree of variance in haplotype expression with only a slight overrepresentation of the less abundant B genotype during infection of wheat, Medicago, and canola (Fig. 4). Homeolog expression dominance has previously been observed between the subgenomes of the uninucleate AG1-IA JN strain (Li et al. 2021). Further analysis of the differentially expressed (DE) homeolog pairs in the JN strain suggested less evolutionary constraint of the DE genes than homeolog pairs with similar expression profiles (Li et al. 2021). This suggested that gene duplication can lead to genetic divergence, potentially acting as a source of variation in pathogenicity and may contribute to the unusually broad host range of AG-8 isolates.

Relatively little is known about the genomic characteristics and pathogenicity genes of disease-causing isolates of *R. solani*. Anderson et al. (2017) compared the secretomes of AG1-1, AG3, and AG-8 isolates and predicted conserved and unique putative secreted effector proteins including a large array of CAZymes but a relatively small number of putative secreted effectors compared to other plant pathogenic fungal species. However, as these genome assemblies were haploid representations of heterokaryotic isolates, the genetic diversity hidden within the homeologs could not be investigated. Similarly, Ghosh et al. (2019) sequenced the genomes of 2 AG1-IA strains highly virulent for causing rice sheath blight in India and compared these to the pre-existing AG1-IA genome. The highly virulent isolates showed expansion or emergence of orthogroups including pectate lyases, glycoside hydrolases, UDP-glucuronosyltransferases, oxidation–reduction-related genes such as NADPH-dependent oxidoreductase, cytochrome P450, and zinc finger transcription factors. Further comparative genomics reported by Liu et al. (2024) identified a protein domain conserved among *R. solani* genome sequences from diverse AGs and only present in basidiomycete species. Silencing of the encoding genes in the AG1-ZJ isolates reduced lesion size on maize and topical RNAi treatment targeting the *R. solani* genes reduced lesion size on maize, rice, and wheat, suggesting the domain may be important for pathogenicity across AGs and potentially on diverse hosts. To expand on this previous work, we assessed the haplotype-specific expression of predicted effectors and annotated CAZymes. In AG8-1 and AG8-3, the 2 haplotypes carry equal proportions of predicted effectors (Table 1), but during infection of wheat, canola, and *Arabidopsis*, the less abundant haplotype B in AG8-1 has a higher proportion of upregulated genes encoding predicted effectors (Fig. 4c). Similarly, the less abundant haplotype A in AG8-3 has a higher proportion of upregulated genes encoding predicted effectors (Fig. 4c). Next, we investigated if the annotated CAZymes exhibit a similar trend. Again, for AG8-3, the less abundant haplotype A has a higher proportion of upregulated genes encoding CAZymes, particularly glycosyltransferases (Fig. 4d). For AG8-1, the less abundant haplotype B has a higher proportion of upregulated genes encoding glycosyltransferases during wheat infection and a higher proportion of upregulated genes encoding carbohydrate esterases during canola infection (Fig. 4d). Taken together, this suggests that both haplotype-specific and host-specific expression patterns play a role during *Rhizoctonia* pathogenesis.

## Conclusion

The generation of high-quality chromosome-level genome assemblies that differentiate haplotypes and enable exploration of the role of these during infection of diverse host crops is an important step to improve crop protection. As a heterokaryotic, multinuclear fungus little was previously known about the diversity between haplotypes in *R. solani* AG-8. Here, we present chromosome-level, nuclear-phased assemblies together with high-quality gene annotations for 2 *R. solani* AG-8 isolates from Australian grain production areas, which are pathogenic on cereals, brassicas, and legumes. This information gives a solid foundation for future research to understand the ecology and epidemiology of *R. solani* AG8 across diverse host genotypes and environments. We anticipate that this new resource will facilitate future discoveries in this pervasive and important fungal pathogen.

## Supplementary Material

jkaf252_Supplementary_Data

## Data Availability

The AG8-1 vegetative and wheat infection RNA-seq reads are available under NCBI BioProject PRJNA371695. The AG8-1 Medicago infection RNA-seq reads are available under NCBI PRJNA369210 and SRP098557. All other data including the genome assemblies, annotations, PacBio Iso-Seq data, and other RNA-seq data are available at the CSIRO Data Access Portal (https://doi.org/10.25919/d5wy-hc17) and under the NCBI BioProject PRJNA1287296. Supplementary Data 1 is also available at the CSIRO Data Access Portal (https://doi.org/10.25919/d5wy-hc17). Supplemental material available at G3 online.
