# Supplementary material for: The fungal pathogen Rhizoctonia solani AG-8 has 2 nuclear haplotypes that differ in abundance
Source: G3 (Bethesda). 2025 Oct 23;16(1):jkaf252. doi: 10.1093/g3journal/jkaf252 (PMC12774589; doi:10.1093/g3journal/jkaf252)
Supplement: jkaf252_Supplementary_Data [file jkaf252_supplementary_data.docx]

**Supplementary Table S1: Genome assembly statistics for *R. solani* AG8-1 and AG8-3 before scaffolding.**

|  | ***R. solani* AG8-1** | ***R. solani* AG8-3** |
| --- | --- | --- |
| Assembly size | 101.895 Mbp | 101.719 Mbp |
| No. of scaffolds | 50 | 61 |
| N50/L50 | 13/3.489 Mbp | 14/2.874 Mbp |

**Supplementary Table S2: Mapping rates for RNA-seq data sets.** Dpi: days post infection.

| **RNA-seq sample** | **Isolate** | **Number of reads** | **% mapped to AG8-1** | **% mapped to AG8-3** |
| --- | --- | --- | --- | --- |
| Arabidopsis 7dpi Replicate 1 | AG8-1 | 96,471,543 | 5.35% | - |
| Arabidopsis 7dpi Replicate 2 |  | 94,072,660 | 2.74% | - |
| Arabidopsis 7dpi Replicate 3 |  | 96,721,875 | 8.84% | - |
| Canola 7dpi Replicate 1 |  | 41,278,768 | 3.31% | - |
| Canola 7dpi Replicate 2 |  | 32,206,582 | 4.31% | - |
| Canola 7dpi Replicate 3 |  | 38,579,162 | 2.96% | - |
| Medicago 7dpi Replicate 1 |  | 90,295,724 | 3.97% | - |
| Medicago 7dpi Replicate 2 |  | 96,589,990 | 3.24% | - |
| Medicago 7dpi Replicate 3 |  | 97,443,323 | 6.64% | - |
| Wheat 7dpi Replicate 1 |  | 35,228,760 | 9.81% | - |
| Wheat 7dpi Replicate 2 |  | 49,137,925 | 8.01% | - |
| Wheat 7dpi Replicate 3 |  | 36,486,393 | 18.95% | - |
| Wheat 7dpi Replicate 1 | AG8-3 | 31,965,766 | - | 6.71% |
| Wheat 7dpi Replicate 2 |  | 45,683,910 | - | 9.78% |
| Wheat 7dpi Replicate 3 |  | 47,054,443 | - | 13.57% |


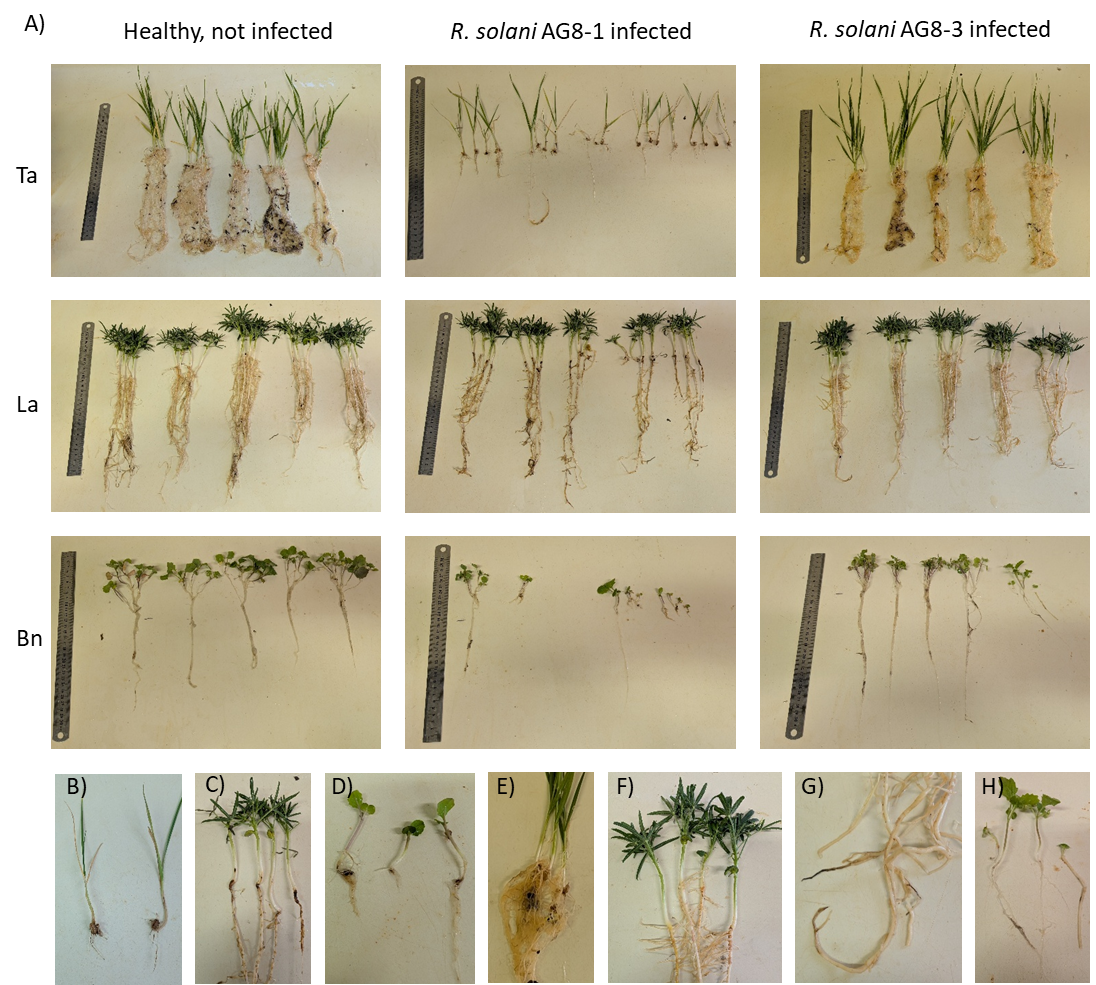


**Supplementary Figure S1**. Symptoms of infection with *R. solani* AG8-1 and AG8-3. A) Healthy, AG8-1 or AG8-3 infected wheat (*Triticum aestivum*, Ta), narrow leaf lupin (*Lupinus angustifolius*, La) and canola (*Brassica napus*, Bn). Symptoms of infection with AG8-1 on wheat (B), narrow leaf lupin (C) and canola (D). Symptoms of infection with AG8-3 on wheat (E), narrow leaf lupin (F, G) and canola (H). For AG8-1 wheat infection short and stubby brown roots and decaying tips (spear tips – pruning of newly emerged roots) and yellowing of leaves is visible (A – Ta; B). For AG8-1 narrow leaf lupin infection short primary lateral and secondary roots with brown and decaying root tips are visible (A-La; C). For AG8-1 canola infection short roots with decaying root tips are visible (A-Bn; D). For AG8-3 wheat infection, very low level of infection with brown roots and some spear tips is visible but overall root growth is not severely affected (A-Ta; E). For AG8-3 narrow leaf lupin infection, some secondary root infection with brown and decaying root tips is present (A-La; F & G) but overall root growth is not severely affected. Lastly, for AG8-3 canola infection minor decaying root tips are visible (A-Bn; H). The length of the ruler is 30 cm. The symptoms from AG8-1 (formerly known as ZG1-1) infection of *M. truncatula* and *Arabidopsis thaliania* are previously described (Perl-Treves et al. 2004; Anderson et al. 2013; Foley et al. 2013).


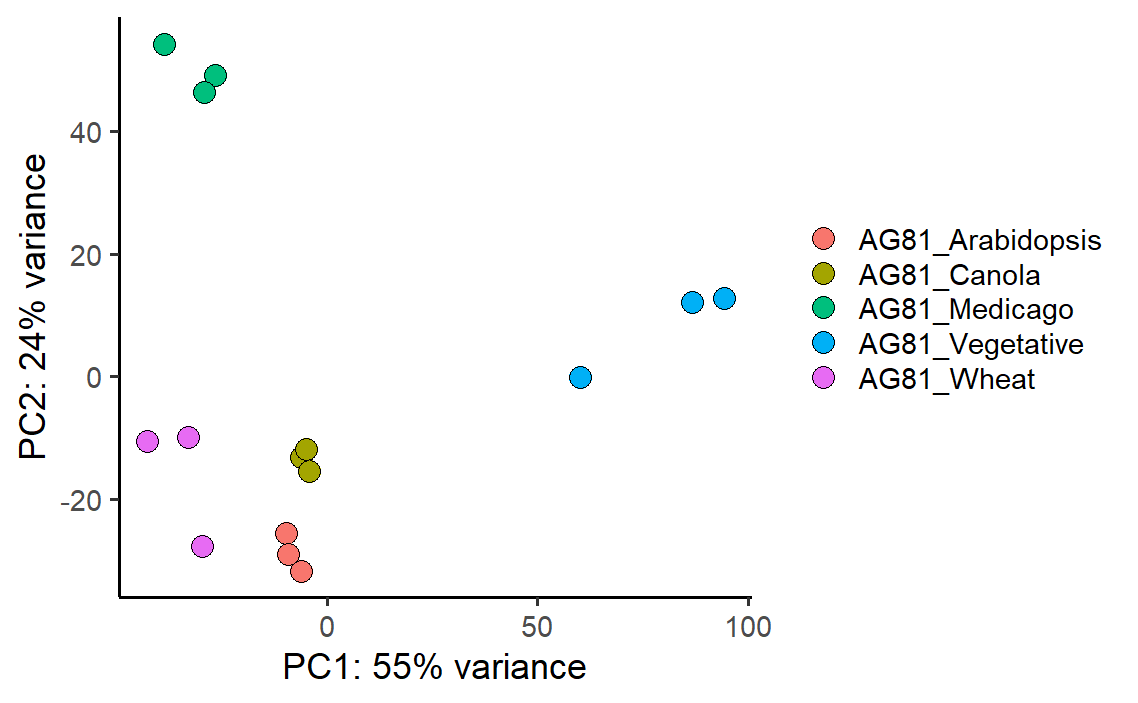
 ~~
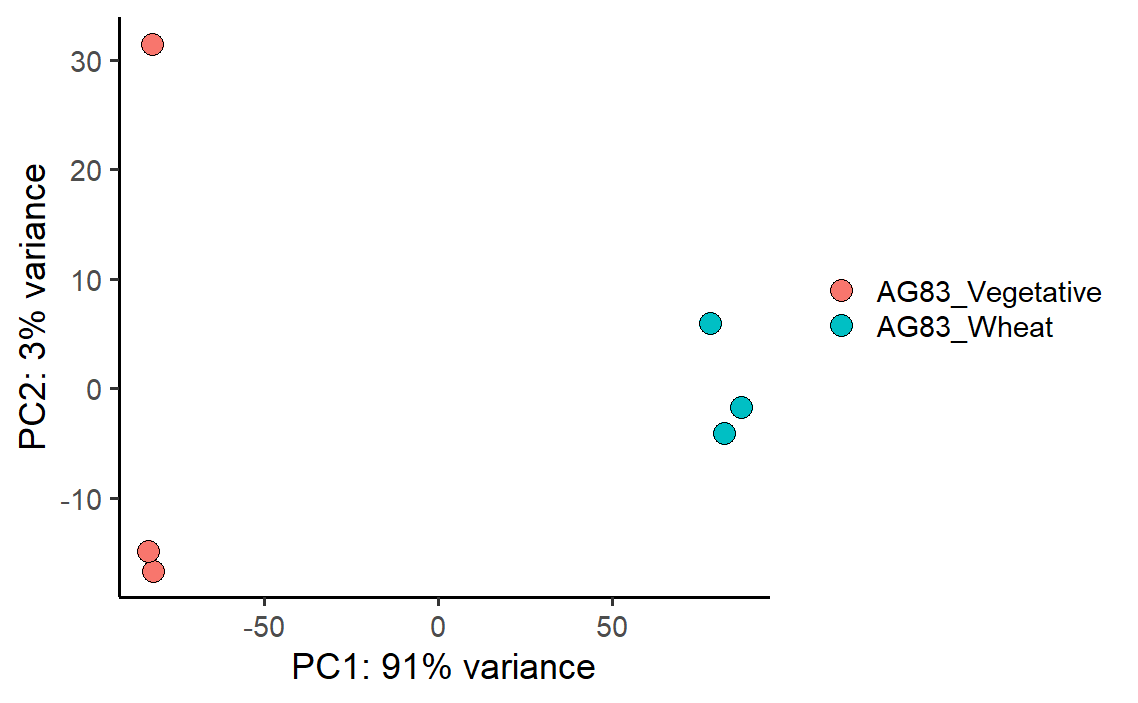
~~

**Supplementary Figure S2: PCA plots for the RNA-seq data sets.**
